# Positron Emission Intensity in the Decay of ^86g^Y for Use in Dosimetry Studies

**DOI:** 10.3390/molecules27030768

**Published:** 2022-01-25

**Authors:** M. Shuza Uddin, Syed M. Qaim, Bernhard Scholten, M. Shamsuzzoha Basunia, Lee A. Bernstein, Ingo Spahn, Bernd Neumaier

**Affiliations:** 1 Institute of Neuroscience and Medicine, INM-5: Nuclear Chemistry, Forschungszentrum Jülich, D-52425 Jülich, Germany; b.scholten@fz-juelich.de (B.S.); i.spahn@fz-juelich.de (I.S.); b.neumaier@fz-juelich.de (B.N.); 2 Tandem Accelerator Facilities, INST, Atomic Energy Research Establishment, Savar, Dhaka 1000, Bangladesh; md.shuzauddin@yahoo.com; 3 Nuclear Science Division, Lawrence Berkeley National Laboratory, Berkeley, CA 94720, USA; sbasunia@lbl.gov (M.S.B.); labernstein@lbl.gov (L.A.B.); 4 Department of Nuclear Engineering, UC Berkeley, Berkeley, CA 94720, USA

**Keywords:** positron emission, electron capture, gamma-ray, X-ray, ^86g^Y radionuclide, matched-pair, theranostic application

## Abstract

The β^+^-emitting radionuclide ^86g^Y (t_1/2_ = 14.7 h) forms a matched-pair with the β^−^-emitting therapeutic radionuclide ^90^Y (t_1/2_ = 2.7 d) for theranostic application in medicine. This approach demands a precise knowledge of the positron emission probability of the PET nuclide which was until recently rather uncertain for ^86g^Y. In this work, an ^86g^Y source of high radionuclidic purity was prepared and a direct measurement of the positron emission intensity per 100 decay of the parent (hereafter “positron emission intensity”) was performed using high-resolution HPGe detector γ-ray spectroscopy. The electron capture intensity was also determined as an additional check by measuring the K_α_ and K_β_ X-rays of energies 14.1 and 15.8 keV, respectively, using a low energy HPGe detector. From those measurements, normalized values of 27.2 ± 2.0% for β^+^-emission and 72.8 ± 2.0% for *EC* were obtained. These results are in excellent agreement with values recently reported in the literature based on a detailed decay scheme study.

## 1. Introduction

Among the various imaging techniques used in diagnostic medicine, the positron emission tomography (PET) occupies a unique position. Since it is based on a coincidence measurement of the two photons generated in the annihilation of a positron in the tissue, it delivers more quantitative results than any other imaging modality. In this regard, the decay properties of the positron-emitting radionuclide affect the overall quality of the tomographic scan. In general, the decay data of the so called “standard” positron emitters, i.e., ^11^C (t_1/2_ = 20.4 min), ^15^O (t_1/2_ = 2.0 min), ^18^F (t_1/2_ = 1.83 h), ^68^Ga (t_1/2_ = 1.13 h), and ^82^Rb (t_1/2_ = 1.3 min) are known well: their positron emission intensity is high, the positron endpoint energy is low and the accompanying γ-rays, if any, do not interfere in the measurement of the annihilation radiation. They are commonly used in patient care to study fast physiological processes, e.g., blood perfusion, oxygen or glucose uptake by brain, etc. However, for studying slow metabolic processes as well as for many novel medical applications, e.g., theranostic approach, bimodal imaging and radionuclide targeted therapy, positron emitters of longer half-lives and different chemistry are needed. Generally, those radionuclides are of metallic nature and are denoted as “non-standard” positron emitters. About 25 such radionuclides have been developed up to the stage of clinical/preclinical application and many other potentially useful ones are under investigation [1,2]. Their use is increasing, but entails three drawbacks: (a) scare availability, (b) rather high positron end-point energy, (c) generally low positron intensity, and (d) associated γ-rays. The last three features affect the resolution in PET imaging and call upon the development of some special algorithms in the analysis of scans, especially for corrections of spurious coincidences between γ-rays and the 511 keV annihilation radiation [3,4]. The low intensity of the emitted positron in comparison to electron capture (EC), especially if not known with high accuracy, may also cause some problem in accurate quantification of PET images.

The availability and status of decay data of positron emitters, especially the positron emission intensities were discussed during several Consultants’ Meetings of the International Atomic Energy Agency (IAEA) [5,6,7] and it was suggested to have a closer look at the decay chains of all the useful and potentially useful positron emitters. It was pointed out that often even the new evaluations are based on older data. A strong recommendation was therefore made to perform new experimental measurements using radioactive samples of high purity. A recent review described the radionuclides for which the reported positron emission intensities are rather uncertain [8].

A new detailed γ-ray spectroscopic measurement was performed on the decay scheme of the standard positron emitter ^82^Rb and, therefrom, the total positron emission probability was deduced [9], which agreed well with the accepted value. Regarding the non-standard positron emitters, new measurements on the direct characterization of the positron emission intensity were carried out for ^45^Ti (t_1/2_ = 3.1 h), ^64^Cu (t_1/2_ = 12.7 h), ^76^Br (t_1/2_ = 16.2 h), ^120^I (t_1/2_ = 1.3 h), and ^124^I (t_1/2_ = 4.2 d) [10,11,12]. The results showed deviations from the existing literature values, thus emphasizing the need of such measurements also on other radionuclides of potential interest.

In this work, we concentrated on the measurement of the positron emission intensity of the non-standard positron emitter ^86g^Y (t_1/2_ = 14.7 h) by determining the ratio of the intensity of the annihilation radiation and of the K X-rays to the decay rate of ^86g^Y. The latter was deduced through a spectroscopic analysis of its four strong γ-rays of well-established intensities. The methodology for production of this radionuclide was developed for PET investigations in 1993 [13,14] at the Forschungszentrum Jülich and the radionuclide was administered to a tumor-bearing patient prior to medication with the beta-emitting therapeutic radionuclide ^90^Y (t_1/2_ = 2.7 d). Based on the distribution kinetics of ^86g^Y, quantitative radiation dosimetry data could be obtained while using ^90^Y [15]. This approach proved to be very successful. Many laboratories worked in this direction; a summary of the progress made is given by Rösch et al. [16]. It is now termed as the theranostic approach and the pair ^86g^Y/^90^Y is called a “matched-pair” of radionuclides. This pair has been used for labelling of antibodies and peptides in many investigations [17,18,19]. In the meantime, several other such pairs are also being considered [20], e.g., ^44g^Sc/^47^Sc, ^64^Cu/^67^Cu, ^124^I/^131^I, etc. Yet the pair ^86g^Y/^90^Y remains in demand. In some very specific therapeutic applications of ^90^Y, the extremely low intensity positron (0.003186%), emitted in its decay via pair-production [21], was used for PET imaging [22,23,24]. For general theranostic applications the use of ^86^Y as the β^+^-emitting partner is more versatile [25]. Furthermore, being a trivalent metal, ^86g^Y is also being considered as an analogue positron emitter for quantification of dose in targeted alpha-particle therapy with the trivalent metal nuclide ^225^Ac [16].

Several reports deal with the decay properties of ^86g^Y [26,27,28]. However, they are all more than 50 years old. The resolution of the detectors used at that time was rather low. In general, this radionuclide decays via electron capture (EC) and β+ emission, followed by emission of a large number of γ-rays. There are six positron groups with different end-point energies and intensities, the sum of which varies between 31 and 34%. According to the latest evaluated decay scheme [29], based on those old data, the γ-rays are fairly well characterized and the summed positron emission intensity amounts to 32.5 ± 2.0%. This value appeared to us rather uncertain. We therefore decided to perform a new measurement via two techniques: (a) high-resolution γ-ray spectroscopy to characterize the 511 keV annihilation radiation and (b) X-ray spectroscopy to determine the EC component. From those measurements the branching ratio β^+^/EC should be deduced. While our work was in progress, Gula et al. [30] reported a detailed spectroscopic analysis of the γ-rays emitted in the decay of ^86g^Y and therefrom deduced the positron emission probability as 27.9 ± 1.2% which is about 14% lower than the evaluated value. Our results obtained via a completely different methodology should then offer a comparison with the results deduced from the most recent experimental decay scheme analysis [30].

## 2. Experimental

The decay parameters for the radionuclide ^86g^Y were determined using activation of an enriched ^86^Sr target provided as ^86^SrCO_3_. The counting methods included γ-ray and X-ray spectroscopy. The salient features of the techniques applied in this work are described.

### 2.1. Target Preparation and Irradiation

Thin strontium carbonate samples were prepared at the Forschungszentrum Jülich (FZJ), Germany, by the sedimentation technique using ^86^Sr-enriched ^86^SrCO_3_ powder (isotopic composition: 96.4% ^86^Sr; 1.33% ^87^Sr; 2.26% ^88^Sr; supplied by Eurisotop, Saint-Aubin, France). Details are given in our earlier study on ^86g^Y-production cross sections [31]. Here, only the important steps are summarized. Al foil of 50-µm thickness and 13-mm diameter (supplied by Goodfellow Cambridge Ltd., Huntingdon, U.K.; chemical purity: 99.0%) was used as the backing material of the sediment. Thereafter, it was covered by a 10-µm thick Al foil of 16-mm diameter welted around the backing foil. The ^86^SrCO_3_ sediment sandwiched between two Al foils then served as the target sample. The areal densities of the two target samples used were 7.614 × 10^−3^ and 7.319 × 10^−3^ g·cm^−2^, where the diameter of each sediment sample was 10 mm. They were irradiated with 8- and 7-MeV protons, respectively, at the BC 1710 cyclotron of FZJ after degradation of the initial proton energy of 17 MeV by degrader foils. Each irradiation was performed for 1 h with a beam current of 250 nA.

From our previous cross-section measurements [31] we estimated that, after the decay of ^86m^Y (t_1/2_ = 47.4 min), the radionuclide ^86g^Y constituted >99% of the activity, the levels of the impurities ^87m,g^Y and ^88^Y being <1%.

### 2.2. Measurement of Radioactivity

#### 2.2.1. γ-ray Spectroscopy

**Figure 1:**
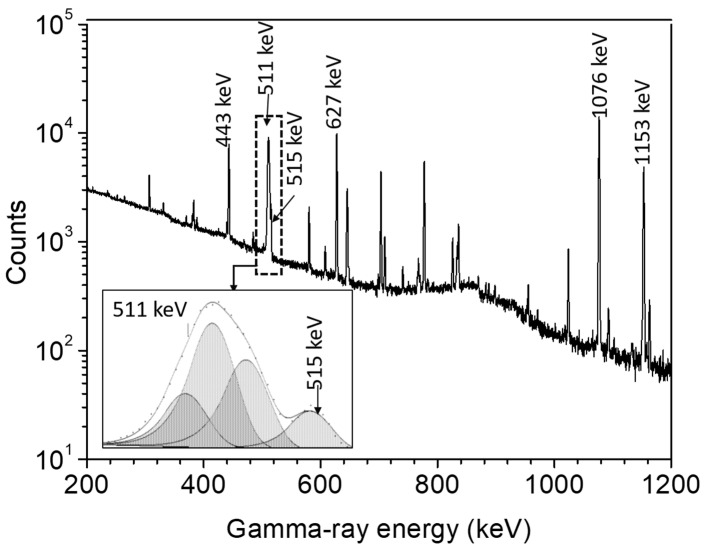
A typical γ-ray spectrum of the enriched ^86^SrCO_3_ sample irradiated with 8-MeV protons: an expanded form of the spectrum around the annihilation peak at 511 keV is given in the inset. The peak is rather broad but it could be quantitatively analyzed.

The radioactivity of the radionuclide ^86^Y was measured non-destructively using two high-purity germanium (HPGe) gamma-ray detectors at FZJ, both supplied by ORTEC. The counting concentrated on the measurement of the ^86g^Y (t_1/2_ = 14.74 h) radioactivity after the complete decay of ^86m^Y (t_1/2_ = 47.4 min) to the ground state. Each detector was associated with the necessary electronics and Maestro data acquisition software. The energy resolution (FWHM) of each detector at 1332.5 keV gamma line of ^60^Co was 1.9 keV. The efficiency calibrations of the detectors were performed using the standard point sources ^22^Na, ^54^Mn, ^57^Co, ^60^Co, ^88^Y, ^137^Cs, ^152^Eu, ^226^Ra, and ^241^Am, supplied by Eckert and Ziegler, Berlin, Germany. The uncertainty in the activity of each standard source was specified as 3%. The γ-ray spectra measured were analyzed by both the GammaVision and FitzPeaks [32] software. Samples were counted at a distance of 20 cm from the surface of the detector, where both the random and true coincidences for the investigated γ-rays were negligible. The dead time of the system was kept below 5%. Each sample was counted repeatedly over several half-lives by giving enough interval to check the radionuclidic purity and to cross-check the results. A typical gamma-ray spectrum for the irradiated target is shown in Figure 1.

Special attention was paid to the determination of the radioactivity of ^86g^Y by measuring the annihilation peak as well as several other γ-rays. In this regard, the following points were important:

(a) Complete annihilation of the emitted positron. Each irradiated ^86^SrCO_3_ sample was placed in a Cu disk with a groove of 13-mm diameter and 1-mm depth, and covered with another Cu disk of the same size. Each Cu disk had a thickness of 5 mm and a diameter of 30 mm, which assured the annihilation of almost all positrons in the decay of ^86g^Y.
(b) A careful analysis of the γ-ray spectrum was carried out to determine the net area under the broad annihilation peak at 511 keV. A part of the neighboring peak at 515 keV, also emitted in the decay of ^86g^Y overlapped the annihilation peak (cf. Figure 1). The net area of the peak at 511 keV, however, could be obtained using the FitzPeak gamma analysis software [32], which was able to isolate the overlapping part. Individual counts of the above two close peaks were also determined by the GammaVision analysis software and the results were comparable to that of the FitzPeak software. The sample was counted several times over two days and the peak area was found to decrease with a half-life of 14.7 ± 0.1 h.

(a) Correction for the in-flight annihilation of the positron. For the positron energies encountered in the decay of ^86g^Y, the net area of the peak at 511 keV in the measured spectrum was increased by a factor of 1.027. This factor was calculated following the method and in-flight annihilation probability in Cu as a function of positron energy of Dolley et al. [33] and the positron spectrum of ^86g^Y obtained using the BetaShape code [34]. It may be mentioned here that the authors at the iThemba LABS in Cape Town [33] also demonstrated in detail that the radioactivity of a source could be quantitatively determined via assay of the annihilation radiation, provided proper precautions are taken.
(b) The natural background around the annihilation radiation was subtracted. A measurement about two weeks and another one about one month after the irradiation were performed to determine some possible background around the annihilation peak from ^87^Y (t_1/2_ = 3.35 d) and ^88^Y (t_1/2_ = 106.6 d), which are expected to be formed in small amounts (<1% of the ^86g^Y activity) via the ^87^Sr(p,n)^87^Y and ^88^Sr(p,n)^88^Y reactions, respectively, on ^87^Sr and ^88^Sr present in low-abundances in the enriched ^86^Sr target used (see Section 2.1). However, no increase above the natural background around the annihilation radiation was observed, obviously due to very weak positron branching (<0.2%) in both ^87^Y and ^88^Y [35].
(c) The contribution to the annihilation radiation through pair production in the interaction of the strong γ-ray of ^86g^Y at 1076.6 keV with the intervening medium was estimated to be negligible.
(d) The presence of other radionuclides which emit radiation in the vicinity of the annihilation radiation, e.g., ^85^Y, ^85^Sr, ^84^Rb, and ^83^Rb, was minimized by using the incident proton energies of 8 and 7 MeV, which are below the thresholds of the proton-induced reactions on ^86^Sr leading to the formation of those radionuclides.
(e) Besides the annihilation peak, four other strong γ-rays emitted in the decay of ^86g^Y were also analyzed using the above-mentioned methodology. Furthermore, their peak areas were corrected for attenuation in Cu, which was determined experimentally by counting each source several times within and without the Cu disks. The averaged radiation transmission factor for many γ-rays of ^86g^Y, except the annihilation line, was deduced. The transmission factor was also calculated using the classical radiation absorption formula. The agreement between experimental and calculated values was excellent.

Based on the above-mentioned experiments and precautions, we could determine the peak areas under the annihilation radiation and the four strong γ-ray peaks of ^86g^Y mentioned above. The decrease in the peak areas of those four specific γ-rays as a function of time also corresponded to a half-life of 14.7 ± 0.1 h. Further treatment of the experimental data is described below.

#### 2.2.2. X-ray Spectroscopy

**Figure 2:**
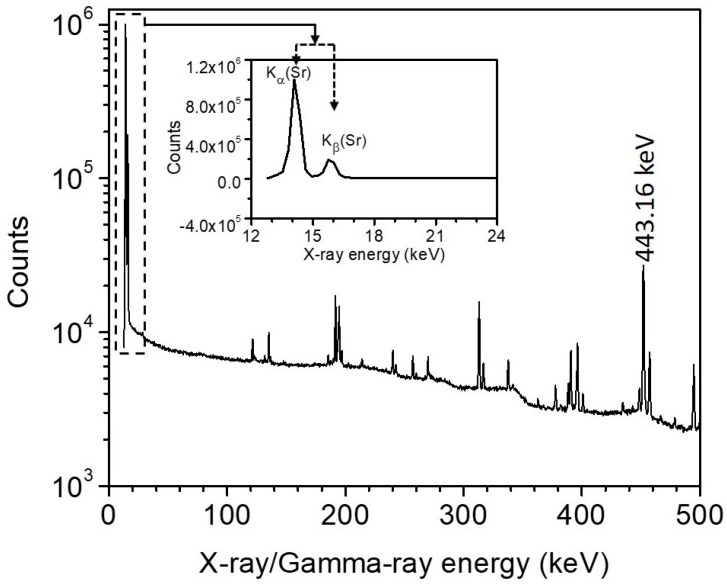
A typical low-energy part of the spectrum of the ^86^SrCO_3_ target irradiated with 8-MeV protons: X-ray peaks below 20 keV (inset) and γ-ray peaks above 100 keV of the product ^86g^Y are discernible.

After the γ-ray spectroscopic measurements on the ^86^SrCO_3_ enriched sample, irradiated with 8- or 7-MeV protons, in which ^86^Y was formed through the ^86^Sr(p,n)-reaction, X-ray spectroscopy was carried out using a special HPGe detector with a thin Be-window of 300-µm thickness, supplied by ORTEC. This detector was especially suited for detection of X-rays and low-energy γ-rays up to 1 MeV. The resolution of the detector (FWHM) was 330 eV at 5.9 keV and 540 eV at 122 keV of ^57^Co. The detector was connected to the necessary electronics and Maestro data acquisition software. For counting, each sample was placed with the 10-µm Al cover facing the detector at 3 cm from the detector surface to obtain good counting statistics. During the measurement the dead time was kept below 3%. Measurements were started about 50 h after the end of bombardment (EOB) and carried out further by giving time intervals long enough to check the half-lives of the activation products. All X-rays from the K-shell, i.e., K_α1–2_ and K_β1–4_, in the energy range of 13.8 to 16.1 keV appeared as two peaks of energies 14.1 and 15.8 keV in the spectrum; they were attributed to Sr which is the product of ^86,87,88^Y decay. The two peaks partially overlapped. A sum of counts for the doublet peak was determined by GammaVision software. The individual net peak area of each peak, however, could be obtained using the FitzPeak gamma analysis software [32], which was able to isolate the overlapping part. A typical spectrum is shown in Figure 2. The two X-ray peaks in the expanded form are shown in the inset.

Two matters were given special consideration in the X-ray spectroscopic measurement.

(a) Assessment of impurity. No X-rays of energies lower than those shown in Figure 2 were observed, suggesting the absence of any Sr or Rb radioisotopes whose decay product would be an element with Z lower than that of Sr. The same result was deduced from γ-ray spectroscopy described above.

A small contribution from the decay of ^87g^Y (t_1/2_ = 80.3 h) was present in the net peak area. This was measured after complete decay of ^86g^Y, and its contribution was estimated by the decay curve analysis. At EOB it amounted to <0.8% of the total count rate. Furthermore, a counting after the ^87g^Y decay suggested that the contribution from ^88^Y was <0.03%. As mentioned above (Section 2.1), the expected contribution of ^87m^Y, ^87g^Y, and ^88^Y was also estimated from the known excitation function of the respective (p,n) reaction and the abundance of the relevant target isotope in the enriched material used. It amounted to about: ^87m^Y (0.4%), ^87g^Y (0.50%), and ^88^Y (0.02%). Those values are comparable to the experimentally determined impurity levels. We therefore placed an upper limit on the impurity level in the ^86g^Y X-ray measurement as 1%.

(b) Efficiency of the detector. The mean energy of all X-rays, derived from K_α_ and K_β_ individual energies and respective intensities, amounted to 14.4 keV. Therefore, the efficiency of the detector at this energy was obtained using a standard ^57^Co source. The advantage is that ^57^Co emits a 14.4 keV γ-ray (intensity: 9.16%), where correction for probability of electron capture from the K-shell was not needed. Nonetheless, the detector efficiency vs. photon energy curve at the counting position was determined using the standard ^57^Co source and four other standard point sources, namely ^93m^Nb, ^210^Pb, ^133^Ba, and ^241^Am, specifically dedicated to X-ray measurements (supplied by Eckert and Ziegler, Berlin, Germany, with uncertainty for each standard as 3%). The fitted efficiency curve gave exactly the same value at 14.4 keV as the individual efficiency obtained from the ^57^Co measurement.

### 2.3. Normalized Count Rate and Estimation of Uncertainties

The peak area (counts) under a characteristic γ-ray as well as an X-ray emitted in the decay of ^86g^Y was converted to count rate and normalized to the end of bombardment (EOB). The count rate was corrected for γ-ray intensity (but not for the annihilation radiation and X-ray which were treated as unknown), efficiency of the detector and absorption in the intervening medium. In the X-ray measurement, corrections were implemented for the fluorescence yield (FY), the probability of electron capture from the K-shell (P_K_), self-absorption in the source, the absorption in the 10 µm Al cover, and for emission of conversion electron from gamma-ray interaction.

From the normalized count rate (CPS), the intensity of the positron emission in the decay of ^86g^Y was calculated using the following Equation (1).

(1) $$\beta^{+}=\frac{{\mathrm{CPS}}_{511\;\mathrm{keV}\;\gamma -\mathrm{ray}}/2\cdot \varepsilon }{{\mathrm{CPS}}_{\gamma -\mathrm{ray}}/I_{\gamma }\cdot \varepsilon }$$

where ε is the detector efficiency at a specific energy and Iγ is the gamma-ray intensity.

The electron capture intensity (EC) was calculated by comparing the partial activity of the X-ray to full activity from the γ-ray using the following Equation (2).

(2) $$\mathrm{EC}=\frac{{\mathrm{CPS}}_{X-\mathrm{ray}}/\varepsilon \cdot \mathrm{FY}\cdot P_{K}}{{\mathrm{CPS}}_{\gamma -\mathrm{ray}}/I_{\gamma }\cdot \varepsilon }$$

Equation (3) was utilized for EC calculation from partial activities of both the X-ray and the annihilation peak.

(3) $$\mathrm{EC}=\frac{{\mathrm{CPS}}_{X-\mathrm{ray}}/\varepsilon \;\cdot \mathrm{FY}\cdot P_{K}}{\left({\mathrm{CPS}}_{X-\mathrm{ray}}/\varepsilon \cdot \mathrm{FY}\cdot P_{K})+({\mathrm{CPS}}_{511\;\mathrm{keV}\;\gamma -\mathrm{ray}}/2\cdot \varepsilon \right)}$$

The combined uncertainty in the positron emission intensity was estimated by taking the square root of the quadratic sum of the individual uncertainties. They were: peak area (0.3–1.5%), correction for annihilation in flight (0.5%), γ-ray intensities (2–3%), and efficiency of the detector (4% for all used γ-rays and 5% for 511 keV). The overall uncertainty for the positron emission intensity amounted to about 7% (1σ). Regarding the intensity of the electron capture decay, again the overall uncertainty was deduced from the square root of the quadratic sum of the individual uncertainties: peak area (0.2–1.4%), X-ray attenuation (2.8%), fluorescence yield (3%), P_K_ value (2%), γ-ray intensities (2–3%), and efficiency of the detector (4% for all used γ-rays and 5% for 511 keV). The overall uncertainty in the measured electron capture intensity amounted to about 6.8–7.2% (1σ).

## 3. Results and Discussion

### 3.1. Intensity of Positron Decay of ^86g^Y

The intensity of positron emission was determined from the ratio of partial activity of the annihilation peak to the full decay rate of ^86g^Y. The latter was determined via four gamma rays of energies and intensities, taken from Gula et al. [30], namely 443.1 keV (16.23%), 627.7 keV (32.58%), 1076.6 keV (82.50%), and 1153.1 keV (30.50%). It should be noted that ^86g^Y emits two close γ-rays at energies of 443.1 keV (15.427%) and 444.1 keV (0.806%) overlapping in the spectrum. Therefore, the total counts of the doublet peak and the sum of their intensities were utilized to determine the activity. The results of the two experiments, described above, are given in Table 1. Each activity value is based on large number of measurements, done at the same detector. The eight individual measured values of the positron intensity were averaged. This resulted in a value of 27.1 ± 1.9%.

### 3.2. Intensity of Electron Capture Decay of ^86g^Y

In order to determine the intensity of EC decay of ^86g^Y, three alternative methods were employed:

(a) Comparison of X-ray and 1076.6 keV γ-ray activities. The X-ray count rate from the low-energy detector was corrected for the detector efficiency, the fluorescence yield (0.69 ± 0.02) [36] and the probability of decay via electron capture from the K-shell (P_K_ = 0.88 ± 0.02) in ^86g^Y decay. The P_K_ was deduced from the calculated electron capture transition branching/intensity, I_EC_, for the ^86^Y decay data set presented in Reference [29] and their corresponding K-shell electron capture probability using the LogFT [37] and BetaShape codes [34]. Both gave consistent results. We also subtracted contribution to K X-ray (~0.5%) from the conversion electrons of the γ-ray transitions in the decay of ^86g^Y. This contribution, resulting from the K-shell electron knock-out by the γ-ray transitions, was estimated using the γ-ray intensities and the conversion coefficient data for known/assumed γ-ray transition multipolarities given by Negret and Singh [29]. On the other hand, the ^86g^Y activity was obtained from the count rate of the most intense 1076.6 keV γ-ray, determined using the well-calibrated detector, and corrected for the efficiency and the intensity. The EC intensity was then determined from the ratio of the X-ray counts to the γ-ray decay rate (Equation (2)).
(b) Comparison of X-ray and 443.1 keV γ-ray activities in the same spectrum. The ^86g^Y activity was determined from the count rate of the 443.1 keV γ-ray corrected for the efficiency and the intensity. It was considered ideal to determine the decay rate of ^86g^Y via the 443.1 keV γ-ray visible in Figure 2 which is based on the use of the same detector for X-rays and this γ-line. However, due to the closeness of the sample to the detector a small correction for the true coincidence summing was necessary. The EC intensity was then determined from the ratio of the above corrected counts of X-ray to the γ-ray decay rate (Equation (2)).
(c) Comparison of X-ray counts to the annihilation peak at 511 keV. In both cases, intensities were not used. The X-ray and 511 keV annihilation photons were counted using different detectors (see above), but the normalized count rate of ^86g^Y in each measurement was extrapolated to end of bombardment (EOB). The normalized count rates thus obtained corresponded practically to the absolute disintegration rates except for correction for the intensity of the counted radiation. The activity obtained from the X-ray was divided by the sum of X-ray and 511 keV annihilation photon, and the EC intensity was calculated using Equation (3). A comparison of the X-ray counts with the 511-keV counts, however, implies that all decay is via K capture and there is either no or only negligible internal conversion.

The results on electron capture intensity obtained via the three techniques are also given in Table 1. The averaged result from the six independent measurements, with the estimated uncertainty, was 72.6 ± 5.2%.

### 3.3. Comparison of Present Results with Literature Data

In this work, the decay of ^86g^Y by positron emission and electron capture is determined to be 27.1 ± 1.9% and 72.6 ± 5.2%, respectively. The normalization of these values yields P_β+_ = 27.2 ± 2.0% and P_EC_ = 72.8 ± 2.0%, respectively, following the procedure of Browne [38]. As discussed above, the results from previous experimental works showed discrepancies, and a later evaluation suggested a value of 32.5 ± 2.0% for the positron intensity. Very recently Gula et al. [30] proposed a new decay scheme of ^86g^Y and estimated that the positron emission amounts to 27.9 ± 1.2%. Our value of 27.2 ± 2.0% obtained through a different experimental technique is thus in complete agreement with that new value.

## 4. Conclusions

The positron emission intensity of ^86g^Y determined in this work experimentally through detailed γ-ray and X-ray spectroscopic analyses of radionuclidically pure sources amounts to a value 27.1 ± 2.0% which is in excellent agreement with a recent value of 27.9 ± 1.2% deduced from an extensive decay scheme study [30]. The new results should strengthen the database for improving the internal dose calculation while using ^86g^Y for PET measurement in theranostic studies together with ^90^Y.

## Figures and Tables

**Table 1:** Measured intensity of positron emission and deduced intensity of electron capture decay of ^86g^Y.

A. Positron Emission				
Experiment	Activity of Annihilation Radiation at EOB (Normalized CPS)	Energy of *γ*-ray (keV)	Decay Rate of ^86g^Y via *γ*-ray * at EOB (DPS)	β^+^ Intensity (%)
1	128,800	443.1	488,801	26.35
		627.7	457,987	28.12
		1076.6	458,362	28.10
		1153.1	464,978	27.70
2	94,889	443.1	365,691	25.95
		627.7	351,112	27.03
		1076.6	356,263	26.63
		1153.1	352,808	26.90
		**Average β^+^ intensity (%): 27.1 ± 1.9 ^††^**		
**B. Electron Capture**				
**Experiment**	**Activity of *X*-ray at EOB (Normalized CPS)**	***EC* Estimated via Annihilation Radiation (%)**	***EC* Estimated via 1076.6 keV *γ*-ray Radiation (%)**	***EC* Estimated via 443.1 keV *γ*-ray Radiation ^†^ (%)**
1	331,601	72.0	72.4	73.5
2	255,296	72.9	71.7	73.3
**Average *EC* (%): 72.6 ± 5.2 ^††^**				

* Adopting *γ*-ray intensities from [30]: 443.1 keV (16.23%), 627.7 keV (32.58%), 1076.6 keV (82.50%), and 1153.1 keV (30.5%). ^†^Activity measured by using a special HPGe detector suited to detect *X*-rays and low-energy *γ*-rays and analyzing the 443.1-keV peak visible in the same spectrum (see Figure 2). ^††^ The deviation depicts the total estimated uncertainty.

## Data Availability

The reported data will be compiled in the international File EXFOR, managed by the IAEA.
